# Challenges from Variation across Regions in Cost Effectiveness Analysis in Multi-Regional Clinical Trials

**DOI:** 10.3389/fphar.2016.00371

**Published:** 2016-10-28

**Authors:** Yunbo Chu, Luyan Dai, Sheng Qi, Matthew Lee Smith, Hui Huang, Yang Li, Ye Shen

**Affiliations:** ^1^Boehringer Ingelheim (China) Investment Co. Ltd.Shanghai, China; ^2^Department of Health Promotion and Behavior, Institute of Gerontology, University of GeorgiaAthens, GA, USA; ^3^Texas A&M School of Public Health, Health Science Center, Texas A&M UniversityCollege Station, TX, USA; ^4^Department of Probability and Statistics, Center for Statistical Science, Peking UniversityBeijing, China; ^5^Center for Applied Statistics and School of Statistics, Renmin University of ChinaBeijing, China; ^6^Department of Epidemiology and Biostatistics, University of GeorgiaAthens, GA, USA

**Keywords:** cost-effectiveness analysis, multi-regional clinical trials, ethnic factors, sensitivity analysis, clinical evidence

## Abstract

Economic evaluation in the form of cost-effectiveness analysis has become a popular means to inform decisions in healthcare. With multi-regional clinical trials in a global development program becoming a new venue for drug efficacy testing in recent decades, questions in methods for cost-effectiveness analysis in the multi-regional clinical trials setting also emerge. This paper addresses some challenges from variation across regions in cost effectiveness analysis in multi-regional clinical trials. Several discussion points are raised for further attention and a multi-regional clinical trial example is presented to illustrate the implications in industrial application. A general message is delivered to call for a depth discussion by all stakeholders to reach an agreement on a good practice in cost-effectiveness analysis in the multi-regional clinical trials. Meanwhile, we recommend an additional consideration of cost-effectiveness analysis results based on the clinical evidence from a certain homogeneous population as sensitivity or scenario analysis upon data availability.

## Introduction

Cost-effectiveness analysis (CEA) involves a comparison of two or more treatments to assess differences in terms of the interventions' costs relative to their effectiveness. One or more new interventions are compared against the intervention currently being implemented. Cost-effectiveness is, by nature, incremental (Weintraub and Cohen, 2009). Thus, the final results of the CEA, accompanied by a proper uncertainty analysis, can be presented as an incremental cost-effectiveness ratio (ICER) and net health benefit (NHB) or net monetary benefit (NMB).

Economic evaluation, in the form of cost-effectiveness analyses, has become a popular means to inform decisions about healthcare priorities (George et al., 2001). It is particularly useful for and applied to decision-makings in the drug market access activities, by comparing ICER to a threshold value, above which an intervention is considered to be not cost effective. Organizations including the National Institute for Health and Care Excellence (NICE) of UK (Birch and Gafni, 2004), Pharmaceutical Benefits Advisory Committee (PBAC) of Australia, and Canadian Agency for Drugs and Technology in Health (CADATH) of Canada require economic evaluations before approving a new drug for market or incorporating a drug into insurance plans (Hjelmgren et al., 2001). When conducting economic evaluation analysis about efficacy and safety outcomes, models are generally created using the data from randomized control trials (RCTs) or they are synthesized using data from meta-analyses. There are a growing number of clinical trials that include data specifically about resource utilization and outcomes for the purposes of cost-effectiveness (Ramsey et al., 2015). However, RCTs do not always provide a sufficient basis for economic evaluations used to inform regulatory and reimbursement decisions (Petrou and Gray, 2011). Usually, the time necessary to perform a cost-effectiveness analysis extends beyond the data collected during RCTs, thus requiring outcome modeling as opposed to direct measurement (Weintraub and Cohen, 2009). To be complete, CEA must consider short-term costs and benefits (e.g., as are observed during an actual RCT) as well as assess longer-term outcomes (Luce et al., 1996). To accomplish this task, decision analysis methodology such as Markov model is often needed to link short—and long-term outcomes.

Final cost-effectiveness results for drugs under appraisal or those that are newly licensed, are often generated by incorporating health outcomes from phase II/III clinical trials with unit costs and healthcare resource utilization of treatment within a specific country. This approach would be straightforward if efficacy/effectiveness data were generated from RCT(s) conducted in a single country where the economic evaluation was conducted. However, multi-regional clinical trials (MRCT) in global development programs have gained popularity in recent decades (Ho and Chow, 1998), especially after the release of the International Council for Harmonisation of Technical Requirements for Pharmaceuticals for Human Use (ICH) Guidance E5 addressing ethnic factors for acceptability of foreign data in the drug registration evaluation (Guideline, 2016a). The rising prevalence of MRCT has introduced new questions about CEA methods, especially surrounding data related to clinical evidence. The economic evidence hereafter refers to resource utilization, unit cost, and their product or a macro costing result.

This paper aims to address common issues encountered when performing cost-effectiveness evaluations of MRCTs with potential heterogeneity and variability across regions. Several discussion points are raised to increase attention about these issues and charge the field to further explore this methodology.

## Heterogeneity in CEA

In general, CEA of a treatment should be analyzed at the country level due to differences in demographics, healthcare systems, and cost structures. Health Technology Assessment (HTA) countries like Australia, Canada, and the UK have established guidelines related to health economic evaluations. Further, an increasing number of other countries have developed principles and recommendations to be used for health economic evaluations (Hjelmgren et al., 2001).

A recent review (Barbieri et al., 2010) documented that the recommendations included within existing guidelines regarding the transferability/variability of clinical and economic data are quite diverse. Almost all guidelines unanimously considered baseline risk and unit costs to be of low transferability, while treatment effects were classified as highly transferable. However, some guidelines recognized the potential differences in the clinical parameters from one country to another, and these country-specific differences can lead to differences in cost-effectiveness estimates.

Theoretically, clinical variability can be attributed to diverse clinical practice patterns or inherent heterogeneity in the patient population within different geographical areas or settings (Canadian Coordinating Office for Health Technology Assessment, 1997). Within a country, the government and other healthcare payers have recognized that a refined reimbursement approach can be achieved to prioritize the patients' needs by gaining a better understanding of the heterogeneity among eligible patients in terms of effectiveness and cost (Coyle et al., 2003). By extension, when a cost-effectiveness analysis based on an MRCT is needed, it may be more appropriate to conduct the analysis within a relative homogeneous group to control the variability in clinical and economic evidences. However, many clinical trials do not have enough subjects in each country/region for clinical effect estimations, and such underpowered subgroup analyses could lead to broader confidence intervals and more uncertainty about CEA results.

To account for variability across different countries/regions in MRCT when conducing CEA, the current popular practice is to use local costs and/or baseline risk, which are associated with high variability. When considering clinical evidences such as treatment effects, overall estimations are often used despite their use presenting challenges in certain circumstances. Using overall estimates may cause confusion by assuming the relative clinical effectiveness of the intervention does not differ across populations. Therefore, we agree with Reed that if the number of enrolled patients is large or there is strong evidence showing the existence of high variability, country—or region-specific clinical evidence, e.g., event rates estimation and treatment effects in response to baseline risks and other local evidences, may be appropriate (Reed, 2012). But, if large patient numbers are not available, modeling CEA with local economic evidences combined with overall clinical evidences would be a plausible trade-off. However, it is still worthy to explore the CEA results based on country—or region specific treatment effects as sensitivity or scenario analyses (e.g., as a reference for local decision makers).

## Consistency within MRCT

Multi-Regional Clinical Trials (MRCT) as a methodology have potential to simultaneously register innovative drugs worldwide. In recent years, efforts have been made for the planning and design of MRCTs to increase the acceptability of MRCTs in global regulatory submissions. ICH is currently drafting the new guidance document E17 to guide the sponsors to address the issues surrounding MRCT (Guideline, 2016b). Well-reasoned and prospectively planned analysis to assess the impact of intrinsic and extrinsic factors on treatment effects are encouraged for the establishment of a good foundation for evaluating the consistency of region specific treatment effects.

With multiple regions involved, intrinsic and extrinsic factors may vary across regions due to ethnicity, culture, patient population, and local medical practice (Guideline, 2016a). Given that the early clinical evidence generally concludes the lack of significant ethnicity-related drug sensitivity and supports regions joining MRCTs, we may not expect these differences to have a major impact on clinical outcomes and treatment effects. Nevertheless, such differences may in fact influence outcomes and effects but not be considered when interpreting the most beneficial treatment for patients.

While the overall treatment effect is addressed and estimated in MRCT, the main challenge is associated with estimating effects within individual regions/countries. This challenge can make it difficult to discern whether signals of regional differences reflect a true interaction or whether the signal might be largely due to statistical “noise” or a chance finding. The subpopulation differences may not be examined in conventional designs using hypothesis testing-based approaches, which rely on the assumption of homogeneity. Conversely, exaggerated signals of differences, which are legitimate concerns if they truly exist, can be expected to arise frequently and might be prone to over-interpretation. Regional differences can be attributed to intrinsic/extrinsic factors (e.g., sociodemographic, medical history, disease severity), and the varying distributions of these factors within countries/regions. While these treatment effects may differ across subpopulations, it is hoped that the variance falls within a fairly narrow range and reasonable degree.

Nevertheless, investigations about such factors are helpful to understand the magnitude of differences to guide recommendations for therapy utilization in clinical practices and assess the associated impact of such therapies on the healthcare costs. Because there is often large variability in data sources with relatively small patient sizes from individual regions/countries, it becomes difficult to draw definitive conclusions solely based on the effect estimation from a particular region/country.

To address this issue, countries can be combined into different definitions of a factor region, and region effects can be compared in different manners. Further, more advanced statistical techniques can be used to identify other similar subpopulations from the overall trial population, which match with the regional subpopulation on key characteristics (Dehejia and Wahba, 2002; Austin, 2014). For instance, recently developed statistical methodologies have suggested that the Bayesian shrinkage estimator can be used for the regional effect estimation based on actual regional results borrowed from other regions (Quan et al., 2013). It considers the treatment effort as a random effort and estimates the mean effect and variance in the region based on the shrinkage.

## A trial example

Dabigatran is an oral direct thrombin inhibitor (DTI) that was approved in recent years for stroke and systemic embolism (SE) prevention among patients with atrial fibrillation (AF). Dabigatran was compared with warfarin in the RE-LY (Randomized Evaluation of Long-Term Anticoagulation Therapy) trial, which was a randomized study comparing two different doses of Dabigatran (i.e., 150 and 110 mg twice a day) with warfarin (Connolly et al., 2009). In total, 18113 patients from 44 countries were included in the trial. After a median 2-year follow-up, the RE-LY trial showed that the time to first stroke or SE (i.e., primary outcome) was significantly better with Dabigatran 150 mg BID than warfarin. This finding was associated with a mean therapeutic time in range of 64.4% (median 67%). Dabigatran 110 mg BID was non-inferior for stroke/SE prevention. Dabigatran 150 mg BID had similar rates of major bleeds, while fewer major bleeds were observed with the Dabigatran 110 mg bid dose (Table 1; Connolly et al., 2009, 2010, 2014).

**Table 1:** **Main outcome in the RE-LY Trial**.

**Variable**	**Dabigatran 110 mg (*N* = 6015)**	**Dabigatran 150 mg (*N* = 6076)**	**Warfarin (*N* = 6022)**	**Dabigatran 110 mg vs. Warfarin**		**Dabigatran 150 mg vs. Warfarin**	
	**Rate/100 person-yr**	**Rate/100 person-yr**	**Rate/100 person-yr**	**Relative Risk (95% CI)**	***P*-value**	**Relative Risk (95% CI)**	***P*-value**
Stroke or SE	1.54	1.12	1.72	0.89 (0.73–1.09)	0.27	0.65 (0.52–0.81)	<0.001
Major bleeding	2.92	3.40	3.61	0.80 (0.70–0.93)	0.003	0.94 (0.82–1.08)	0.41
Myocardial infarction	0.82	0.81	0.64	1.29 (0.96–1.75)	0.09	1.27 (0.94–1.71)	0.12
Gastrointestinal major bleeding	1.15	1.56	1.07	1.08 (0.85–1.38)	0.52	1.48 (1.18–1.85)	0.001

To assess the cost-effectiveness of Dabigatran, models were developed to quantify comparisons with current care for stroke prevention among patients with AF. Patients with AF were followed for their remaining lifetime using model-based approaches (e.g., Markov model). Treatment patterns and costs were reflective of each country's healthcare system. Risk of clinical events for patients on warfarin and Dabigatran were based on observed event rates in the RE-LY trial.

**Figure 1:**
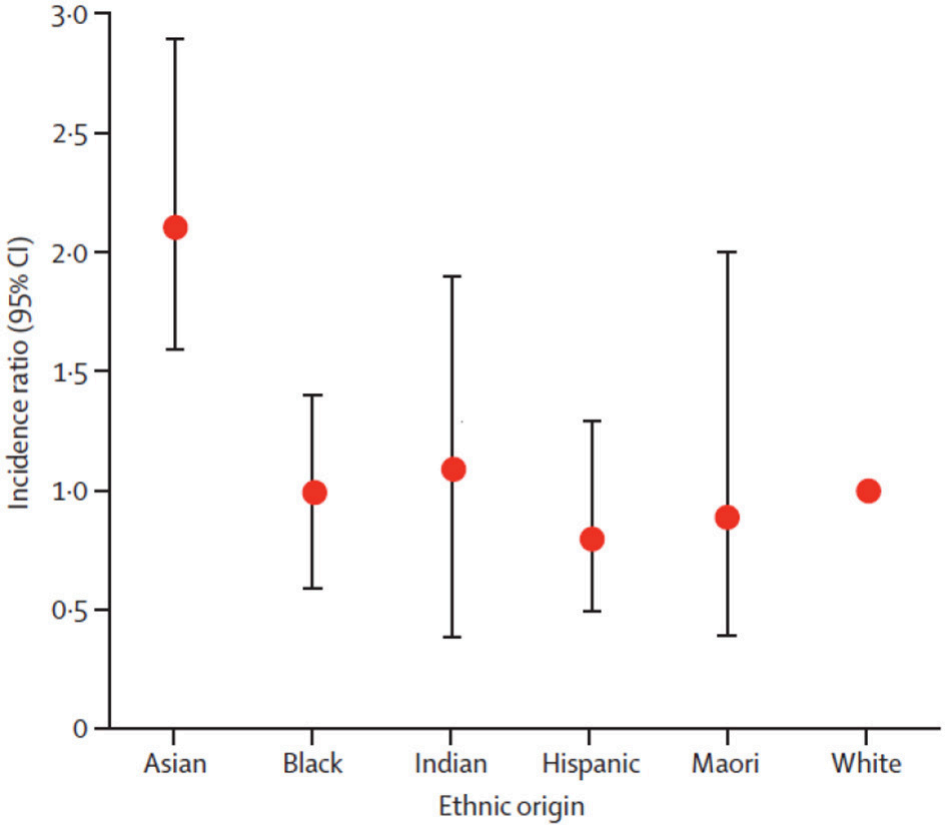
**Intracerebral hemorrhage incidence ratios in ethnic groups**. White ethnic origin was taken as reference. Circles are means and bars are 95% CI. Reprinted from van Asch et al. (2010), Copyright (2010), with permission from Elsevier

However, in some cases, the events rates in different country/region subgroups might be inconsistent with the overall population (for reasons previously described). For instance, the incidence of intracerebral hemorrhage among Asian patients was higher relative to patients of other ethnic origins (Figure 1). The incidence of intracerebral hemorrhage among East and Southeast Asian patients (51.8 per 100 000 person-years, 95% CI 38.8–69.3) was over twice the incidence for Caucasian patients (24.2, 95% CI 20.9–28.0, reference group) (van Asch et al., 2010). Furthermore, evidence shows that patients of Asian ethnicity are also at a greater risk of hemorrhage while receiving vitamin K antagonist therapy (Shen et al., 2007).

**Figure 2:**
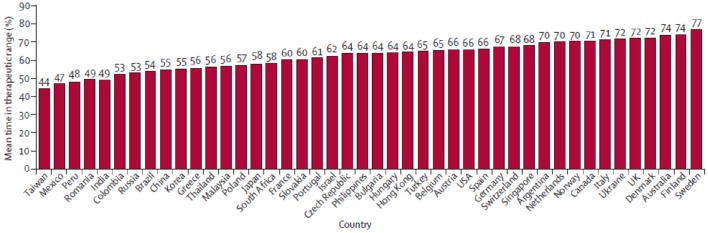
**Country distribution of mean time in therapeutic range in the RE-LY trial**. Reprinted from Wallentin et al. (2010), Copyright (2010), with permission from Elsevier.

Another outcome of interest from the RE-LY trail was time in therapeutic range (TTR), which is a commonly used measure of the adequacy of international normalized ratio (INR) control in studies with a warfarin arm. Increasing levels of site TTR are associated with reduced rates of thrombotic events among patients with AF treated with warfarin. But the warfarin control was not balanced by region. In general, TTR was higher in Western Europe and North America and lower in some locations in the Asia-Pacific basin (Figure 2; Wallentin et al., 2010). This variability of TTR will change the hazard ratio in warfarin controlled trials of novel anticoagulants.

There were 2782 patients (15%) from 10 Asian countries in the RE-LY trial. TTR was lower among Asian patients (mean, 54.5%) relative to non-Asian patients (66.2%), with less time above (10.1% in Asian vs. 14.0% in non-Asian) and more time below the therapeutic range (35.4 vs. 19.8%). As further noticed in sub-group analyses, although Asian patients on warfarin had considerably more time below therapeutic range and were younger, a trend for more bleeding among Asian patients was observed when compared to non-Asian patients. Meanwhile, Dabigatran reduced the risk of bleeding outcomes more among Asians than in non-Asians. There was a significant interaction between treatment and region (Asian vs. non-Asian). The effects of Dabigatran against stroke and SE were similar among Asian and non-Asian patients for both doses of Dabigatran when compared with warfarin (Table 2; Hori et al., 2013).

**Table 2:** **Main outcomes with Dabigatran vs. Warfarin in Asians in RE-LY trial**.

	**Dabigatran 110 mg (*N* = 923)**	**Dabigatran 150 mg (*N* = 933)**	**Warfarin (*N* = 926)**	**Dabigatran 110 mg vs. Warfarin**	**Dabigatran 150 mg vs. Warfarin**
	**%/y**	**%/y**	**%/y**	**HR (95% CI)**	**HR (95% CI)**
Stroke or SE	2.50	1.39	3.06	0.81 (0.54–1.21)	0.45 (0.28–0.72)
Major bleeding	2.22	2.17	3.82	0.57 (0.39–0.85)	0.57 (0.38–0.84)
Myocardial infarction	0.51	0.50	0.58	0.88 (0.36–2.17)	0.87 (0.35–2.13)
Gastrointestinal major bleeding	1.15	0.96	1.41	0.82 (0.45–1.49)	0.69 (0.37–1.27)

From the results of the RE-LY trial and its Asian subgroup analysis, the efficacy of Dabigatran on stroke/SE was consistent between Asian and non-Asians; however, the reductions in major and total bleeding were greater with Dabigatran compared to warfarin among Asian patients. The occurrence of MI in Asian patients was also lower compared with that of non-Asian patients. The emerging evidence suggests that if we use the observed events rates from the RE-LY Asian sub-group, together with the typical economic evidence from Asian countries' healthcare systems, a more suitable CEA would be possible for local decision-makers' reference.

## Concluding remarks

The frequency of using MRCT will continue to increase despite the difficulties associated with previously published cost-effectiveness studies. One challenge is associated with the quality of treatment effects data, which is crucial to CEA. Variability is concerned with situations where input parameters may vary systematically between recipients or locations, and thus such variance should be considered and adjusted for in MRCT. Though similar topics have been raised in terms of heterogeneity, variability, and transferability in economic evaluations using MRCT (Willke et al., 1998; Reed et al., 2005; Drummond et al., 2009), such reports have not clearly stated that the CEA methods associated with MRCT varied between studies. In this context, we suggest this issue be further discussed in-depth by stakeholders in the scientific community to reach an agreement about recommended best practices. Until then, especially given increasing demands region/country-specific results to guide local healthcare decisions, we recommend that CEA include sensitivity or scenario analyses among certain homogeneous subpopulations (as upon data availability) to improve clinical decision making for diverse patients.

## Author contributions

All authors contributed extensively to the work presented in this paper including substantial contribution to the design/concept presented, analysis, and interpretation. All authors have been involved in crafting and editing drafts, have approved the final submitted version, and agree to be accountable for all aspects of the work.

## Funding

YL research is supported by the Fundamental Research Funds for the Central Universities from the Ministry of Education of the People's Republic of China, and the Research Funds (15XNI011) of Renmin University of China.

### Conflict of interest statement

The authors declare that the research was conducted in the absence of any commercial or financial relationships that could be construed as a potential conflict of interest.
